# Carnosine inhibits carbonic anhydrase IX-mediated extracellular acidosis and suppresses growth of HeLa tumor xenografts

**DOI:** 10.1186/1471-2407-14-358

**Published:** 2014-05-22

**Authors:** Zuzana Ditte, Peter Ditte, Martina Labudova, Veronika Simko, Filippo Iuliano, Miriam Zatovicova, Lucia Csaderova, Silvia Pastorekova, Jaromir Pastorek

**Affiliations:** 1 Department of Molecular Medicine, Institute of Virology, Slovak Academy of Sciences, Dubravska cesta 9, Bratislava 845 05, Slovak Republic; 2 Center for Molecular Medicine, Slovak Academy of Sciences, Vlarska 3-7, Bratislava 831 01, Slovak Republic

**Keywords:** Carbonic anhydrase IX, Hypoxia, Carnosine, pH regulation

## Abstract

**Background:**

Carbonic anhydrase IX (CA IX) is a transmembrane enzyme that is present in many types of solid tumors. Expression of CA IX is driven predominantly by the hypoxia-inducible factor (HIF) pathway and helps to maintain intracellular pH homeostasis under hypoxic conditions, resulting in acidification of the tumor microenvironment. Carnosine (β-alanyl-L-histidine) is an anti-tumorigenic agent that inhibits the proliferation of cancer cells. In this study, we investigated the role of CA IX in carnosine-mediated antitumor activity and whether the underlying mechanism involves transcriptional and translational modulation of HIF-1α and CA IX and/or altered CA IX function.

**Methods:**

The effect of carnosine was studied using two-dimensional cell monolayers of several cell lines with endogenous CA IX expression as well as Madin Darby canine kidney transfectants, three-dimensional HeLa spheroids, and an *in vivo* model of HeLa xenografts in nude mice. mRNA and protein expression and protein localization were analyzed by real-time PCR, western blot analysis, and immunofluorescence staining, respectively. Cell viability was measured by a flow cytometric assay. Expression of HIF-1α and CA IX in tumors was assessed by immunohistochemical staining. Real-time measurement of pH was performed using a sensor dish reader. Binding of CA IX to specific antibodies and metabolon partners was investigated by competitive ELISA and proximity ligation assays, respectively.

**Results:**

Carnosine increased the expression levels of HIF-1α and HIF targets and increased the extracellular pH, suggesting an inhibitory effect on CA IX-mediated acidosis. Moreover, carnosine significantly inhibited the growth of three-dimensional spheroids and tumor xenografts compared with untreated controls. Competitive ELISA showed that carnosine disrupted binding between CA IX and antibodies specific for its catalytic domain. This finding was supported by reduced formation of the functional metabolon of CA IX and anion exchanger 2 in the presence of carnosine.

**Conclusions:**

Our results indicate that interaction of carnosine with CA IX leads to conformational changes of CA IX and impaired formation of its metabolon, which in turn disrupts CA IX function. These findings suggest that carnosine could be a promising anticancer drug through its ability to attenuate the activity of CA IX.

## Background

Hypoxia in the tumor microenvironment is associated with poor prognosis and a poor response to therapy, underlying the importance of studying the effect of potential anticancer drugs on the hypoxia pathway. Stabilization of hypoxia-inducible factor 1 (HIF-1) as an adaptive response to hypoxic conditions in tissues results in transcriptional activation of many genes that play an important role in cancer-related processes, such as angiogenesis, cell survival, glucose metabolism, and cell invasion. HIF-1 is a heterodimer consisting of a constitutively expressed HIF-1β subunit and a HIF-1α subunit that is regulated through O_2_-dependent degradation modulated by prolyl hydroxylation. The von Hippel–Lindau (VHL) tumor-suppressor protein binds specifically to hydroxylated HIF-1α which is then ubiquitylated by E3 ubiquitin-protein ligases and rapidly degraded by the proteasome [1].

The dipeptide β-alanyl-L-histidine, also known as carnosine, was described for the first time in the 19^th^ century [2]. Carnosine is naturally present in cardiac and skeletal muscles and the central nervous system, and is synthesized from β-alanine and L-histidine by carnosine synthase in muscle cells, glial cells, and oligodendrocytes [3]. Carnosine plays a role as a physiologic pH buffering substance and antioxidant [4]. It induces variable effects on the cardiovascular system, including down-regulation of blood pressure [5,6], inhibition of glycosylated low-density lipoprotein formation [7], and inhibition of angiotensin-converting enzyme activity [8]. It also acts as an anti-aging agent [9]. Moreover, it inhibits proliferation of cells derived from patients with glioblastoma [10] and the growth of tumors formed from neoplastic cell lines, such as Sarcoma-180 tumor cells [11], various neoplastic human and rodent cell lines [12], cells expressing the human epidermal growth factor receptor 2 (Her2/neu) [13], and HCT116 colon cancer cells [14]. Conversely, carnosine enhances the proliferation potential of cultured normal human fibroblasts, lengthens their lifespan, and suppresses senescence [9]. The mechanism of its action in tumor cells remains unclear.

Proteomic studies of glioblastoma cells after treatment with carnosine revealed significantly reduced expression of von Hippel-Lindau binding protein 1 (VBP1) [15], a protein that binds to the von Hippel-Lindau protein [16] and thus is linked to HIF-1α signaling. Pretreatment with carnosine reduced the induction of HIF-1α protein in H9c2 cardiomyoblasts during hypoxia and further reduced its already low level under normoxia; the level of HIF-1 mRNA was transiently reduced after carnosine treatment, but increased after 24 h in a similar manner to controls. A similar experiment with human astrocytes showed that carnosine did not significantly alter the pattern of HIF-1α protein expression in these cells [17].

Carbonic anhydrase IX (CA IX) is a membrane-bound metalloenzyme that is expressed in a broad range of solid tumors [18]. The main function of CA IX is to maintain intracellular pH homeostasis under hypoxic conditions that are common in solid tumors although it also modulates E-cadherin-mediated cell adhesion via its interaction with beta-catenin, which could be of potential significance in hypoxia-induced tumor progression [19]. CA IX contributes to ion transport and pH control by forming a bicarbonate transport metabolon with the sodium bicarbonate transporter NBCe1 and anion exchanger 2 (AE2) [20,21]. CA IX expression in tumors is recognized as a marker of hypoxia and an indicator of poor prognosis. Moreover, CA IX possesses clinical potential as a target for anticancer treatment [22]; indeed, functional inhibition of CA IX has been proposed as an attractive option for therapeutic targeting of various hypoxic tumors [18]. Transcription of the gene encoding CA IX is primarily activated by the hypoxia-inducible HIF-1 transcription factor that binds to the hypoxia response element (HRE) located next to the transcription initiation site [23]. Phosphorylation of Thr443 of CA IX by protein kinase A (PKA) in hypoxic cells is critical for its activation [21].

Because kinetic and X-ray crystallographic studies suggest that carnosine is a potent activator of the carbonic anhydrase isoforms hCA I, II, and IV [24] and the studies described above indicate that carnosine affects the HIF-1 signaling pathway, we initially examined whether CA IX is involved in the antitumor activity of carnosine. We subsequently investigated whether carnosine exerts its effect on CA IX through modulation of transcription and translation levels of HIF-1α and CA IX and/or through altering CA IX function.

## Methods

### Cell culture and spheroid preparation

Madin-Darby canine kidney (MDCK), HeLa, HT-29, and SiHa cell lines were obtained from the American Type Culture Collection and cultured in Dulbecco’s modified Eagle’s medium (DMEM) supplemented with 10% fetal calf serum (FCS; Bio Whittaker) and gentamicin (Sandoz) at 37°C and 5% CO_2_ in humidified air. The cells were counted, seeded in 3- or 6-cm Petri dishes (Greiner) for 24 h, and treated with L-carnosine (Sigma Aldrich) under normoxic (incubator, 5% CO_2_) and hypoxic (2% O_2_, 2% H_2_, 5%CO_2_, 91% N_2_, anaerobic workstation, Ruskinn Technology) conditions.

HeLa spheroids were generated by seeding cells (1,250 cells/well) in 96-well plates (Greiner) coated with 1% agarose. After 4 days of incubation at 37°C and 5% CO_2,_ the spheroids were photographed and treatment was initiated by addition of fresh medium with or without carnosine. In all experiments, at least 30 replicate wells were set up for the control and the carnosine treatment groups. Photographs were taken every 48 h. At the end of the experiment, extracellular pH was measured and the spheroids were subjected to flow cytometric analysis to determine cell viability.

### Measurement of extracellular pH using sensor dish reader

The sensor dish reader (SDR; PreSense) monitors pH in real-time in special plates (HydroDish®) using a non-invasive technique that detects the luminescence lifetime of a sensor spot at the bottom of each well that is dependent on the pH of the surrounding sample. Cells were seeded into wells and allowed to attach. Measurement was started on the second day, when the cells reached 80% confluence. Cells were cultured in the presence or absence of carnosine under hypoxic or normoxic conditions as described above. The pH was measured by the SDR every 30 min.

### Competitive ELISA

HeLa cells were cultured in 96-well plates for 24 h in normoxic conditions and then in hypoxic conditions for additional 24 h, followed by 6-h treatment with different concentrations of carnosine (1.531, 3.0625, 6.125, 12.25, 25, 50, 100 mM) with or without specific antibodies against different domains of the CA IX protein (MAb10, MAb12) [25]. Cells were fixed with methanol, blocked with 10% FCS in phosphate-buffered saline (PBS) for 30 min, and incubated with HRP-conjugated secondary antibody for 1.5 h at room temperature. Absorbance and color changes were measured at 492 nm.

### Immunofluorescence (IF)

HeLa cells grown on glass coverslips were fixed in methanol. After blocking in 3% bovine serum albumin (BSA)/PBS, the cells were incubated with primary antibodies against CA IX (M75 hybridoma medium) or against HIF-1α (diluted in PBS with 0.5% BSA) for 1 h at 37°C. The cells were washed four times for 10 min with PBS containing 0.02% Tween 20, incubated for 1 h at 37°C with Alexa-conjugated secondary antibody diluted in PBS with 0.5% BSA, and washed three times with PBS. All experiments were also performed in the absence of the primary, secondary, or both antibodies as negative controls. Nuclei were stained with 4',6-diamidino-2-phenylindole (DAPI; 1:36000; Sigma Aldrich) for 5 min. Finally, the cells were mounted in Fluoroshield Mounting Medium (Abcam) and analyzed by laser scanning microscopy (LSM 510 Meta Microscope; Zeiss).

To investigate the influence of carnosine treatment on the binding of fluorescein isothiocyanate (FITC)-labeled CA specific inhibitor (FITC_CAI), HeLa cells were cultured without and with 20 mM carnosine in normoxic and hypoxic conditions. After 48 h, the medium was replaced by fresh medium containing FITC_CAI at a final concentration of 0.1 mmol/L. After further incubation for 1 h, the live cells were analyzed by laser scanning microscopy (LSM 510 Meta Microscope; Zeiss) using the incubation stage set at 37°C and 5% CO_2_. FITC-labeled carbonic anhydrase specific inhibitor (FITC_CAI) [26] was a gift from Professor C.T. Supuran.

### Proximity ligation assay

The proximity ligation assay (PLA) was used for *in situ* detection of the interaction between CA IX and AE2. The assay was performed in a humid chamber at 37°C according to the manufacturer’s instructions (Olink Bioscience). SiHa cells were seeded on glass coverslips and allowed to attach before transfer to 2% hypoxia and further cultured for 24 h. After starvation overnight in DMEM supplemented with 0.5% FCS, carnosine was added to selected samples (final concentration 20 mM) and the control and treated cells were cultured for an additional 24 h in hypoxia. The cells were fixed with methanol, blocked with 3% BSA/PBS for 30 min, incubated with a mixture of antibodies against CA IX and AE2 for 1 h, washed three times, and incubated with plus and minus PLA probes for 1 h. The cells were washed, incubated with ligation mixture containing connector oligonucleotides for 30 min, washed again, and incubated with amplification mixture containing fluorescently labeled DNA probe for 100 min. After a final wash, the samples were mounted and the signal representing interaction between CA IX and AE2 was analyzed using a Zeiss LSM 510 Meta confocal microscope.

### Flow cytometry analysis (FACS)

HeLa cells were treated with carnosine (5–40 mM) under normoxic and hypoxic conditions. After 48 h, the cells were detached using trypsin, which was then inactivated by 10% FCS in PBS with 2 mM EDTA. Cells were centrifuged and resuspended in PBS with 10% FCS at a final concentration of 1 × 10^6^ cells/mL. For measurement of the surface expression of CA IX protein, 100 μL of hybridoma medium containing a M75 antibody against CA IX was added to 100 μL of the sample. After incubation at 4°C for 30 min, the cells were centrifuged, washed twice with PBS, and incubated with the secondary Alexa Fluor 488 donkey anti-mouse antibody. Cells stained with only secondary antibody were used as a negative control. For assessment of cell viability, the cells were stained with propidium iodide at a final concentration of 5 μg/mL and incubated for 5 min at room temperature. The samples were analyzed using a Guava EasyCyte Plus flow cytometer with Guava Express Pro 2.2.3 software (Millipore).

### Western blotting

For western blotting (WB), cells grown in confluent monolayers were rinsed twice with cold PBS, resuspended in ice-cold lysis buffer (1% Triton X-100; 50 mM Tris pH 7,5; 150 mM NaCl; 0,5% Nonidet P-40) containing protease (Roche) and phosphatase inhibitors cocktail (Sigma Aldrich), disrupted by sonication and cleared by centrifugation. Protein concentrations were quantified using the BCA protein assay reagents (Pierce). Protein extracts (100 μg/lane) were resolved in 8% SDS-PAGE and transferred to a polyvinylidene difluoride (PVDF) membrane (Macherey-Nagel). The total level of CA IX protein was detected by HRP-conjugated M75 antibody, and HIF-1α and actin were detected using purified primary antibodies and the appropriate HRP-conjugated secondary antibodies as described in the section Antibodies. Protein bands were visualized using an enhanced chemiluminescence kit (GE Healthcare Bio-Sciences).

### Real-Time quantitative PCR (qPCR)

HeLa cells were cultured with or without 20 mM carnosine in normoxia and hypoxia for 48 h. Total RNA was isolated using Instapure solution (Eurogentech) and reverse transcription of RNA was performed with the High-Capacity cDNA Reverse Transcription kit (Applied Biosystems) according to the manufacturer’s recommendations. Amplification was performed in a Stratagene Mx 3005P thermal cycling block (Agilent Technologies). PCR was carried out in 20-μL volumes using Maxima Syber Green PCR Master Mix (Fermentas) for 10 min at 95°C for initial denaturation followed by 40 cycles of 95°C for 15 s and 60°C for 1 min. Sample Ct values were normalized to actin. Relative expression was calculated using the ΔΔCt method. All amplifications were performed in triplicate in three independent experiments. Oligonucleotides used for real-time qPCR were as follows: *HIF-1α sense* 5′-GCTTGGTGCTGATTTGTGAACC-3′, *HIF-1α antisense* 5′-GCATCCTGTACTGTCCTGTGGTG-3′, *Actin sense* 5′-CCAACCGCGAGAAGATGACC-3′, *Actin antisense* 5′-GATCTTCATGAGGTAGTCAGT-3′*, GLUT-1 sense* 5′-CTCCTTTCTCCAGCCAGCAATG-3′, *GLUT-1 antisense* 5′-CCAGCAGAACGGGTGGCCATAG-3′, *VEGF sense* 5′-CAGCACGGT CCCTCTTGGAA-3′, *VEGF antisense* 5′-CCTCCTCTTCCCTGTCAGGA-3′, *VBP1 sense* 5′-CTGTGGTTGGGGGCTAATGT-3′, *VBP1 antisense* 5′-CCCTGGCCATATTGACTTCTGT-3′.

### Chromatin immunoprecipitation (ChIP)

HeLa cells were plated onto 10-cm Petri dishes, cultured to approximately 70% monolayer density, and then incubated in the presence or absence of 20 mM carnosine in hypoxic conditions for additional 48 h. The cells were fixed in 1% formaldehyde directly in medium at room temperature (~21°C) for 15 min. Chromatin isolation and immunoprecipitation with antibody against HIF-1α were performed using Exacta ChIP (R&D Systems) according to the manufacturer’s instructions. DNA was purified using the Wizard SV Gel and PCR Clean-Up System (Promega). Amplification of the samples was performed with HF Phusion polymerase (Thermo Fisher) in an automatic DNA thermal cycler (Eppendorf) using initial denaturation at 98°C for 9 min followed by 43 cycles of denaturation at 97°C for 1 min and annealing at 62°C for 1 min. Primers flanking the HRE elements within the *CA9* and *VEGF* promoter were as follows: *hCA9 HRE sense* 5′-TCCTAGCTTTGGTATGGGGGAGAG-3′, *hCA9 HRE antisense* 5′-AGTGACAGCAGCAGTTGCACAGT-3′, *hVEGF HRE sense* 5′-CCTCAGTTCCCTGGCAACATCTG-3′, *hVEGF HRE antisense* 5′-CCTCAGTTCCCTGGCAACATCTG-3′.

### Animal experiments

CD1 nude mice were purchased from Charles River Laboratories. The animals had access to standard food and water *ad libitum*. Ten male animals were injected subcutaneously into the flank on both sides with 2 × 10^6^ HeLa cells in 100 μL sterile PBS. At 14 days after implantation, the animals were divided into two groups: the first group (five animals) was treated with carnosine (50 μL of 1 M stock, dissolved in sterile PBS) administered subcutaneously 2 cm from the implantation site every second day, and the second group (five animals) was used as a control (sterile PBS only). Tumor size was determined by caliper measurements and was calculated according to the formula W^2^ *L/2, where W is the width and L the length of the tumor. All animal protocols were approved by the Institutional Ethics Committee of the Institute of Virology and the State Veterinary and Food Institute of the Slovak Republic (Permit no. 4245/13-221).

### Immunohistochemistry (IHC)

Tumor specimens were fixed in formalin, dehydrated in an ethanol series, treated with xylene, and mounted in paraffin. Serial sections of tissues were cut and deparaffinized in a xylene and ethanol series. Immunostaining for HIF-1α was performed after antigen retrieval (125°C for 5 min, 95°C for 10 min in citrate buffer, pH 6) using the Dako Cytomation Catalyzed Signal Amplification System kit (Dako). CA IX staining was performed using Dako EnVision + System-HRP (Dako). Cell nuclei were counterstained with hematoxylin solution.

### Antibodies

*Primary antibodies:* mouse monoclonal anti-human HIF-1α (dilutions used: WB 1:250, IF 1:150, IHC 1:150; BD Transduction Laboratories); goat polyclonal anti-human actin (WB 1:1000; Santa Cruz Biotechnology); rabbit anti-human AE2 (PLA 1:500) [20]; mouse monoclonal anti-human carbonic anhydrase IX-M75 hybridoma medium (PLA, FACS, IF non-diluted; IHC 1:100); purified mouse monoclonal anti-human carbonic anhydrase IX–MAb10, MAb12 (ELISA 200 μg/mL) [25]; purified M75 antibody against CA IX conjugated with HRP (WB 1:7500).

*Secondary antibodies:* Alexa Fluor 488-conjugated donkey anti-mouse IgG (IF 1:1000, FACS 1:3000; Invitrogen); HRP-conjugated goat anti-mouse IgG (WB 1:5000; Dako); HRP-conjugated rabbit anti-goat IgG (WB 1:5000; Dako).

## Results

### Carnosine reduces CAIX-mediated acidification

**Figure 1:**
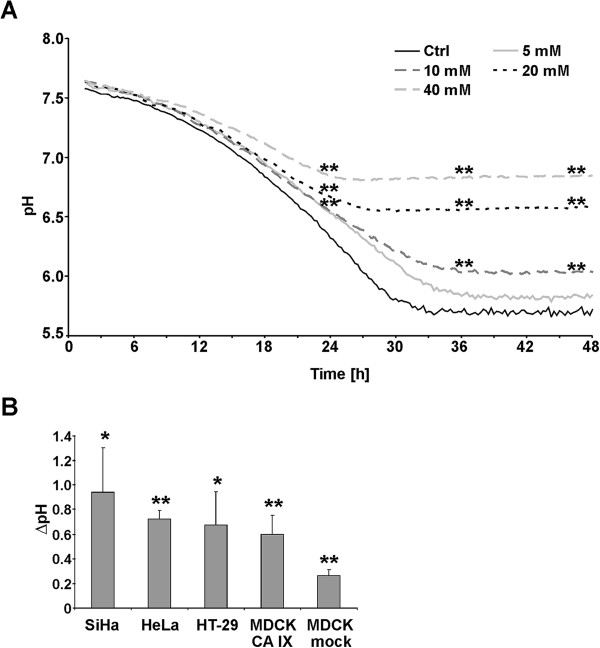
**Carnosine decreases acidification of the growth media of different cell lines. (A)** HeLa cells were incubated for 48 h in hypoxic conditions in the presence of different concentrations of carnosine (5–40 mM) and the extracellular pH was measured by SDR in real time. Carnosine treatment markedly decreased the acidification of growth media in a dose-dependent manner. **denotes p < 0.01 for the comparison between samples treated with different carnosine concentrations and the untreated control. **(B)** SiHa, HeLa, HT-29, MDCK-CA IX, and MDCK-mock cells were exposed to hypoxia for 48 h in the presence of 20 mM carnosine. The columns in the graph represent differences in the endpoint pH values of carnosine-treated cells and their respective untreated controls. Carnosine caused considerable alkalization of growth media in all studied cell lines, except for MDCK mock cells in which the effect of carnosine was noticeably lower. Differences between endpoint pH values of the untreated control and carnosine-treated cells were evaluated by a t-test (*p < 0.05, **p < 0.01).

Cultivation of HeLa cells under hypoxia for 48 h in the presence of carnosine (5–40 mM) resulted in reduced acidification of the extracellular environment in a dose-dependent manner (Figure 1A). The effect of carnosine on HeLa cells in normoxic conditions was substantially smaller (data not shown). Because of its physiologic relevance, a carnosine concentration of 20 mM was selected for further tests on different cancer cell lines (SiHa, HeLa, HT-29). Incubation with carnosine markedly reduced the acidification of growth media in hypoxic conditions for all cell lines studied (Figure 1B, Additional file 1). We also observed a carnosine-mediated decrease in acidification in MDCK cells transfected with CA IX, whereas the effect of carnosine on their mock-transfected counterparts was considerably smaller.

### Effect of carnosine on the level of total CA IX

**Figure 2:**
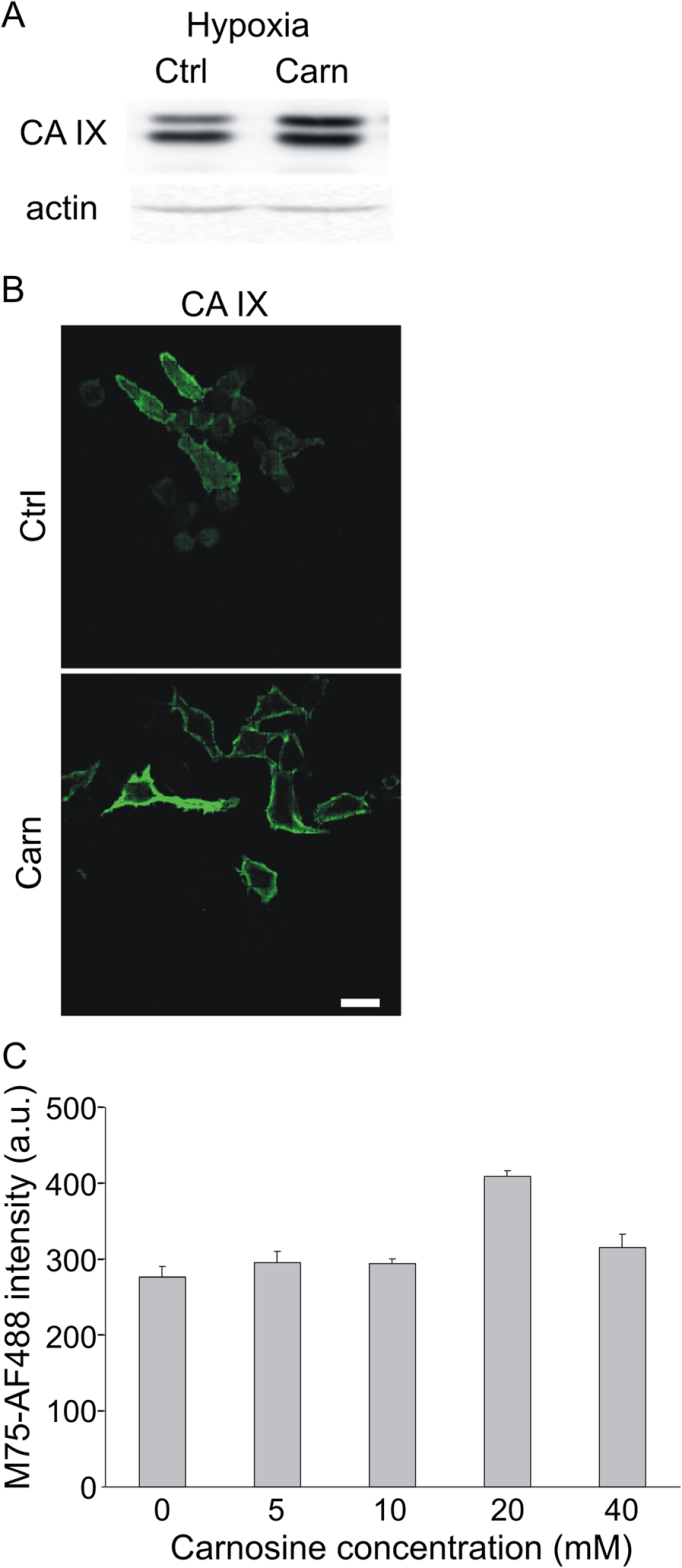
**Effect of carnosine on the level of CA IX protein.** HeLa cells were cultured in hypoxic conditions for 48 h in the presence of 20 mM carnosine. Carnosine induced a slight increase in CA IX protein level in hypoxic cells as shown by western blot analysis **(A)** and immunofluorescence (scale bar 20 μm) **(B)**. This result was confirmed by flow cytometric analysis using M75 antibody against CA IX **(C)**. After treatment with 20 mM carnosine, the level of membrane-localized CA IX increased by 48% compared with the control. Other concentrations of carnosine did not have a comparable effect on CA IX protein level. Differences between the untreated control and carnosine-treated samples were evaluated by a t-test (*p < 0.05).

To determine whether the carnosine-mediated reduction in extracellular acidification of CA IX-positive cells is related to CA IX protein level, we cultivated HeLa cells in hypoxic conditions and used our in-house anti-CA IX antibody M75 to measure CA IX protein levels. The level of CA IX protein increased after carnosine treatment (Figure 2A). This result was confirmed by immunofluorescent staining of CA IX (Figure 2B) and by flow cytometry analysis, which showed that 20 mM carnosine treatment increased the levels of surface CA IX in HeLa cells under hypoxia (Figure 2C). Carnosine did not change the degree of phosphorylation at Thr443, suggesting that it has no effect on activation of CA IX through phosphorylation by PKA (Additional file 2).

### Carnosine treatment increases the level of HIF-1α protein and mRNA and the expression of hypoxia-regulated genes

**Figure 3:**
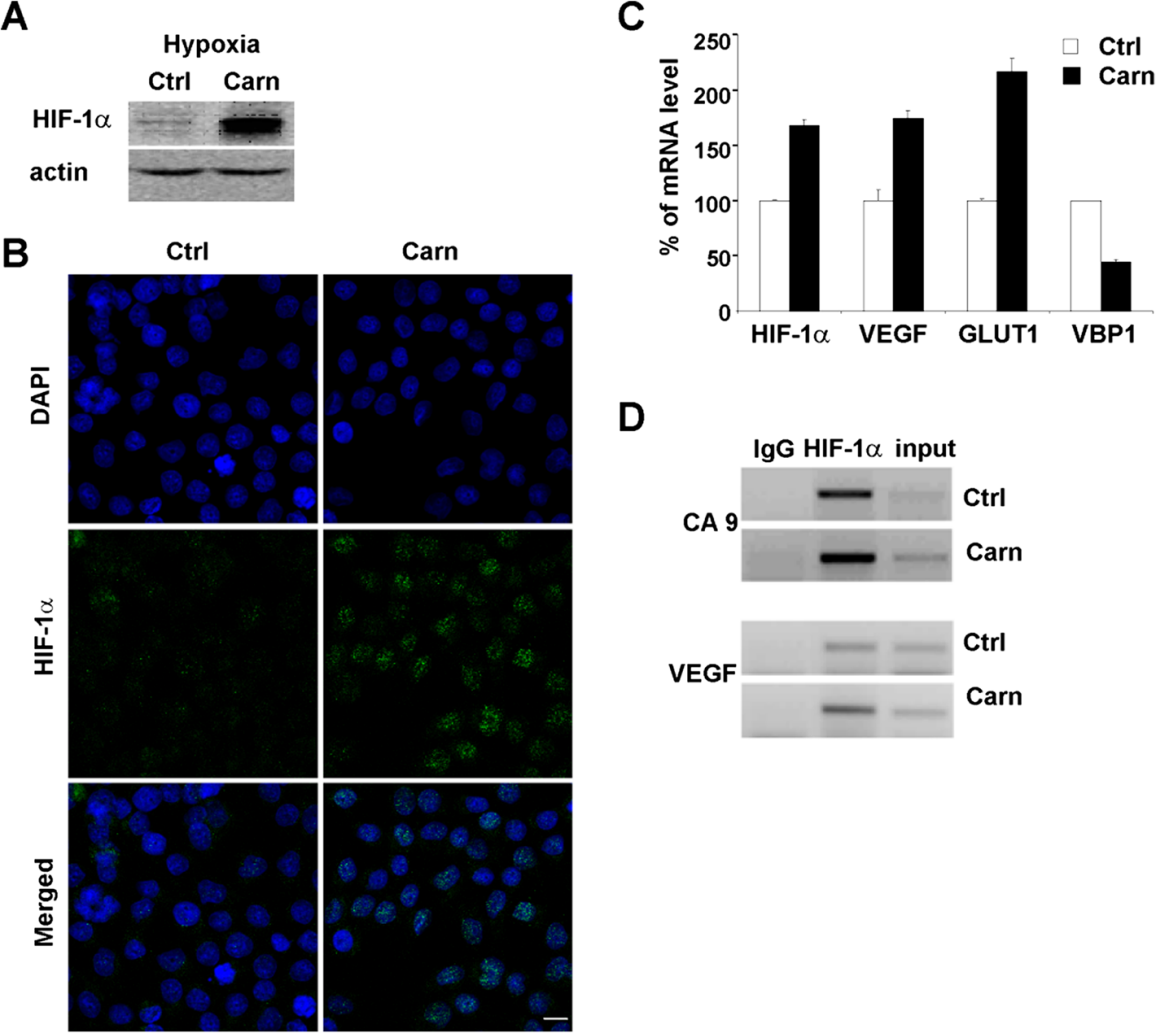
**Effect of carnosine on the level of HIF-1α.** HIF-1α protein level was increased in hypoxic HeLa cells cultured in the presence of 20 mM carnosine for 48 h as demonstrated by western blot **(A)** and immunofluorescence (scale bar, 10 μm) **(B)**. **(C)** Carnosine treatment increased expression of *HIF-1α* mRNA and its target genes *VEGF* and *GLUT-1* under hypoxic conditions. Concurrently, the mRNA level of *VBP1*, a protein that binds to VHL and is involved in HIF-1α degradation, was decreased. **(D)** Chromatin immunoprecipitation assay performed under the same conditions demonstrated that HIF-1α bound to hypoxia-responsive elements in the promoter region of *VEGF* and *CA9* genes.

Because transcription of CA IX is activated by HIF-1α, we tested whether carnosine influenced HIF-1α protein and mRNA levels in HeLa cells. HeLa cells were cultured in hypoxic conditions for 48 h with or without 20 mM carnosine. Western blot analysis showed a significant increase in HIF-1α signal in cells treated with carnosine compared with controls (Figure 3A). This finding was supported by immunofluorescent staining of HIF-1α, which showed a stronger HIF-1α signal in the nuclei of treated cells (Figure 3B). Data from qPCR analysis confirmed an increased level of *HIF-1α* mRNA after carnosine treatment under hypoxia compared with the untreated control (Figure 3C). The activity of HIF-1α was demonstrated by the increase in mRNA expression of the HIF-1α targets vascular endothelial growth factor (VEGF) and glucose transporter 1 (GLUT-1) after carnosine treatment in hypoxia (Figure 3C). Moreover, ChIP analysis showed a moderate increase in binding of HIF-1α to the HRE in both *CA9* and *VEGF* promoters (Figure 3D). Interestingly, the level of *VBP1* mRNA decreased after carnosine treatment compared with the control (Figure 3C), indicating reduced degradation and increased stabilization of HIF-1α protein.

### Carnosine inhibits binding of CA-specific inhibitor and CA IX-specific antibodies and impairs formation of the CA IX metabolon

**Figure 4:**
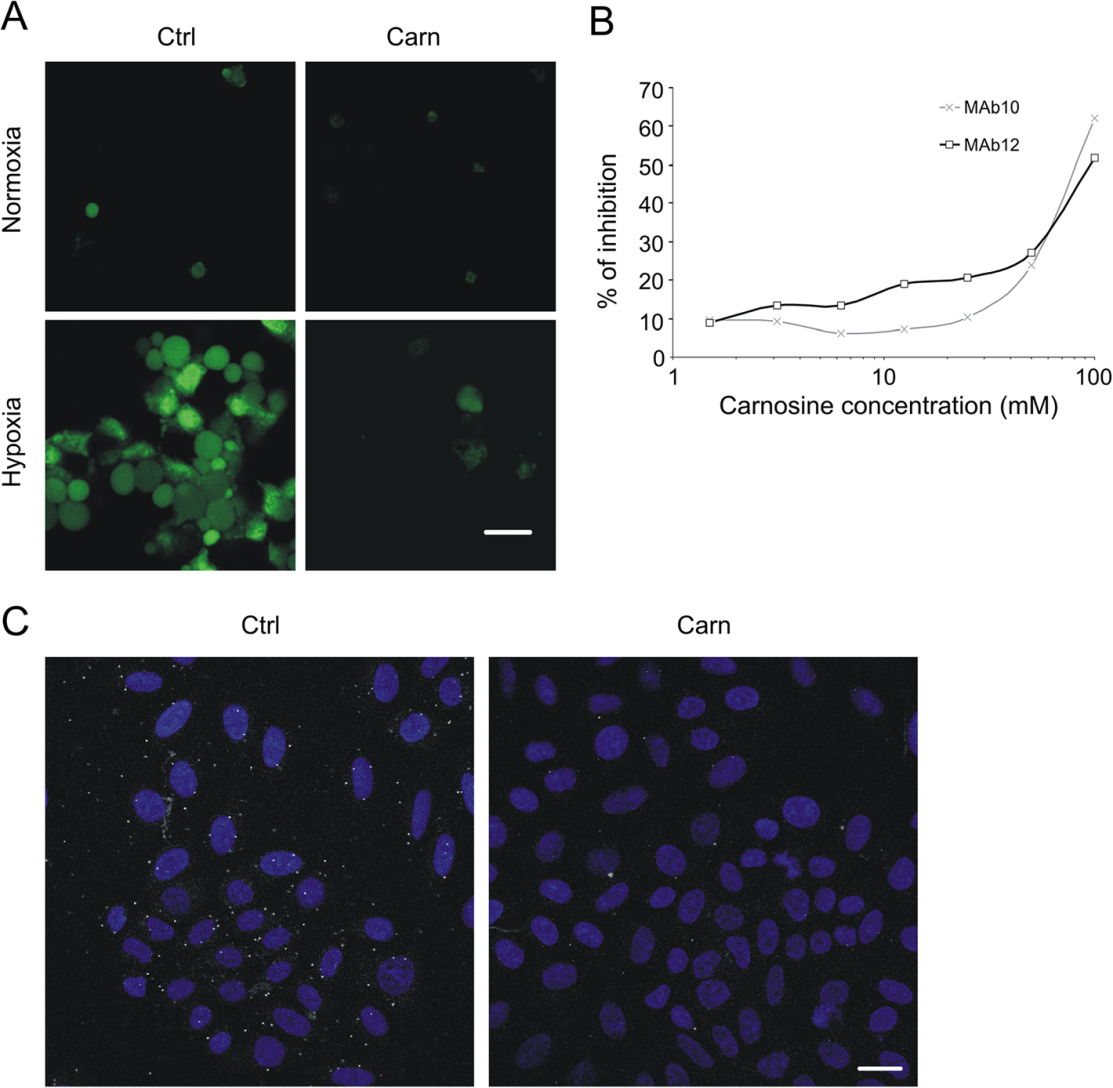
**Carnosine impairs binding of CA IX-specific inhibitor and antibodies and affects formation of a CA IX metabolon. (A)** Immunofluorescence signal of FITC-labeled CA specific inhibitor, which binds only to hypoxic cells with catalytically active CA IX, was markedly reduced after treatment of HeLa cells with 20 mM carnosine in hypoxia for 48 h. **(B)** Graph of competitive inhibition ELISA showing the percentage of inhibition after cultivation of HeLa cells with different concentrations of carnosine together with specific CA IX antibodies MAb10 and MAb12. Binding of antibodies to conformational epitopes in the catalytic domain of CA IX was inhibited by carnosine in a concentration-dependent manner. **(C)** SiHa cells cultured in islands under hypoxic conditions were analyzed by proximity ligation assay to visualize direct interaction between CA IX and the anion exchanger AE2 in the functional metabolon. The positive signal represented by bright white dots was considerably reduced in cells treated with 20 mM carnosine for 24 h, supporting the proposal that binding of carnosine alters the conformation of CA IX. Scale bar, 20 μm.

We next investigated binding of fluorescein-conjugated CA-specific homosulfanilamide inhibitor (FITC_CAI) to carnosine-treated and untreated cells in hypoxic conditions. Svastova *et al*. previously showed that FITC_CAI binds only to hypoxic cells expressing CA IX, and it is widely accepted that this inhibitor binds only to catalytically active CA IX that has been activated by hypoxia [26]. We observed a reduction in the immunofluorescent signal of FITC-CAI after carnosine treatment of HeLa cells (Figure 4A) and MDCK-CA IX cells (data not shown) under hypoxia, indicating a decrease in CA IX activity in the presence of carnosine. This assumption is supported by the results of competitive inhibition ELISA performed in HeLa cells after culture in the presence of different concentrations of carnosine together with the CA IX-specific antibodies MAb10 and MAb12 directed against conformational epitopes in the catalytic domain of CA IX. As shown in Figure 4B, carnosine inhibited the binding of MAb10 and MAb12. Furthermore, a proximity ligation assay showed that carnosine treatment reduced the signal arising from direct interaction of CA IX and AE2 in the metabolon of SiHa cells (Figure 4C).

### Carnosine treatment reduces spheroid size and cell viability

**Figure 5:**
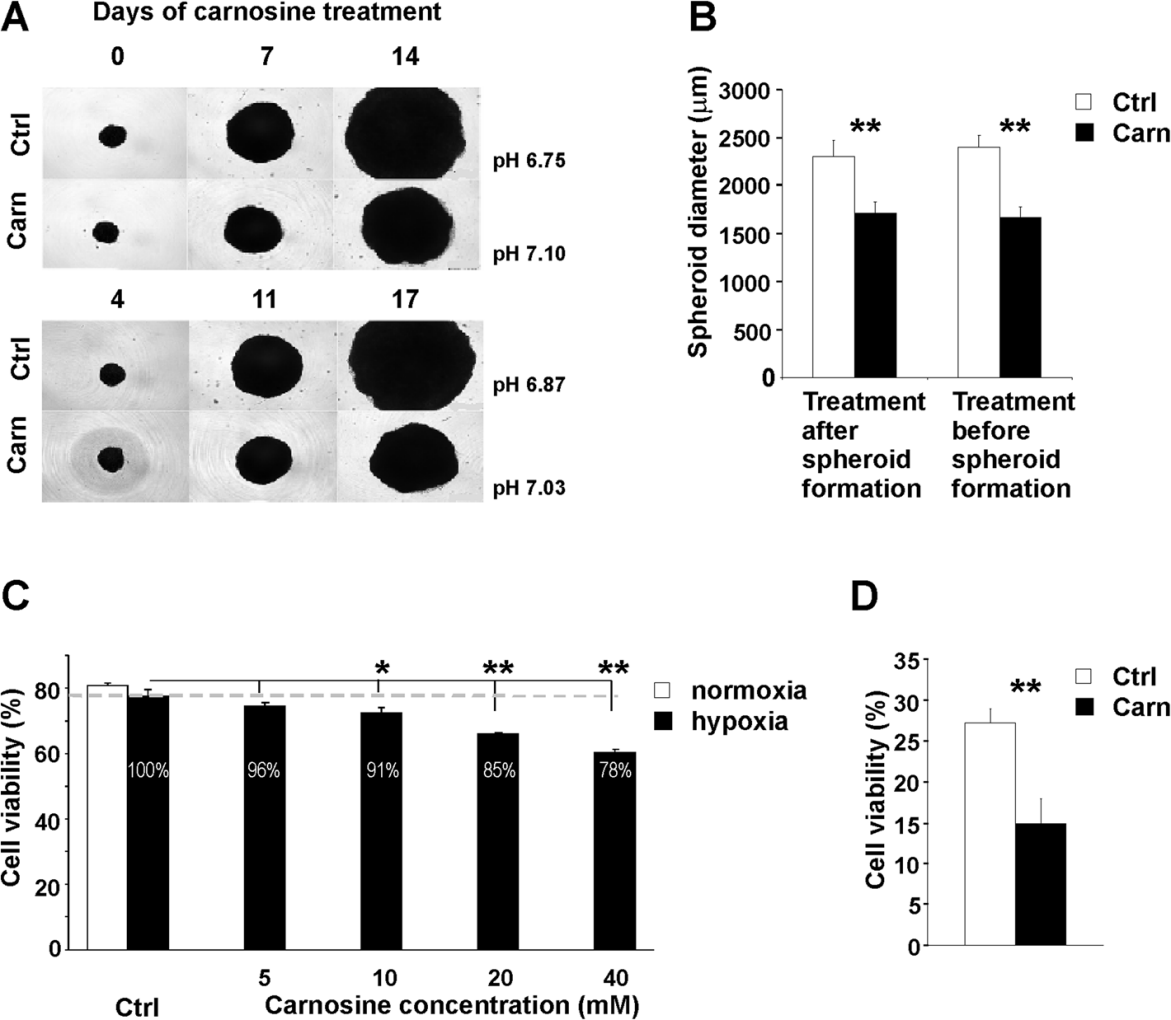
**Effect of carnosine on spheroid size and cell viability. (A)** Images of spheroids cultured in the presence and absence of 20 mM carnosine. Carnosine was added after spheroids had already formed (upper image) or before spheroid formation (lower image). The images show the same time points of spheroid growth, although the duration of carnosine treatment differs as indicated, depending whether carnosine was added before or after spheroid formation. pH values represent measured pH of media collected from all respective spheroid samples (N > 30) at the end of the experiment. **(B)** In both experimental conditions, the diameters of carnosine-treated spheroids were significantly smaller than those of controls (t-test, **p < 0.01). **(C)** Flow cytometry showed that treatment with different concentrations of carnosine (5–40 mM) decreased cell viability in hypoxic conditions in a dose-dependent manner (t-test, *p < 0.05, **p < 0.01). Numbers in the columns give the ratio for the viability of a carnosine-treated sample and the respective hypoxic control (set as 100%). The ratio of the viability of hypoxic and normoxic controls was 96%. **(D)** Cultivation of HeLa spheroids in the presence of 20 mM carnosine significantly reduced cell viability by approximately 50% compared with the control group (t-test, **p < 0.01).

To confirm the effect of carnosine in a physiologically more relevant three-dimensional (3D) environment, we treated spheroids formed by HeLa cells with carnosine added to the culture medium only after the spheroids had already formed, or with carnosine present during the period of spheroid formation. Both experimental groups formed spheroids, indicating that spheroid formation was not significantly affected by carnosine. At the end of the experiment the carnosine-treated spheroids in both groups had a significantly smaller (almost 50% smaller) diameter than the controls; moreover, the extracellular pH of the treated groups was higher in the treated cultures than in the controls (Figure 5A, 5B).Data from flow cytometric analysis showed that carnosine treatment of a two-dimensional monolayer culture decreased the viability of hypoxic cells in a dose-dependent manner: 5 mM carnosine decreased HeLa cells viability only slightly, 10 mM carnosine by approximately 10%, and 20 mM by approximately 15% (Figure 5C). In comparison, the viability of HeLa cells in normoxic conditions remained relatively constant in the presence of different concentrations of carnosine (data not shown). In 3D culture, where hypoxia develops in the center of spheroids, we observed a marked decrease in viability of HeLa spheroids of 50% after treatment with 20 mM carnosine compared with the controls (Figure 5D).

### Carnosine reduces tumor size in an experimental mouse xenograft model

**Figure 6:**
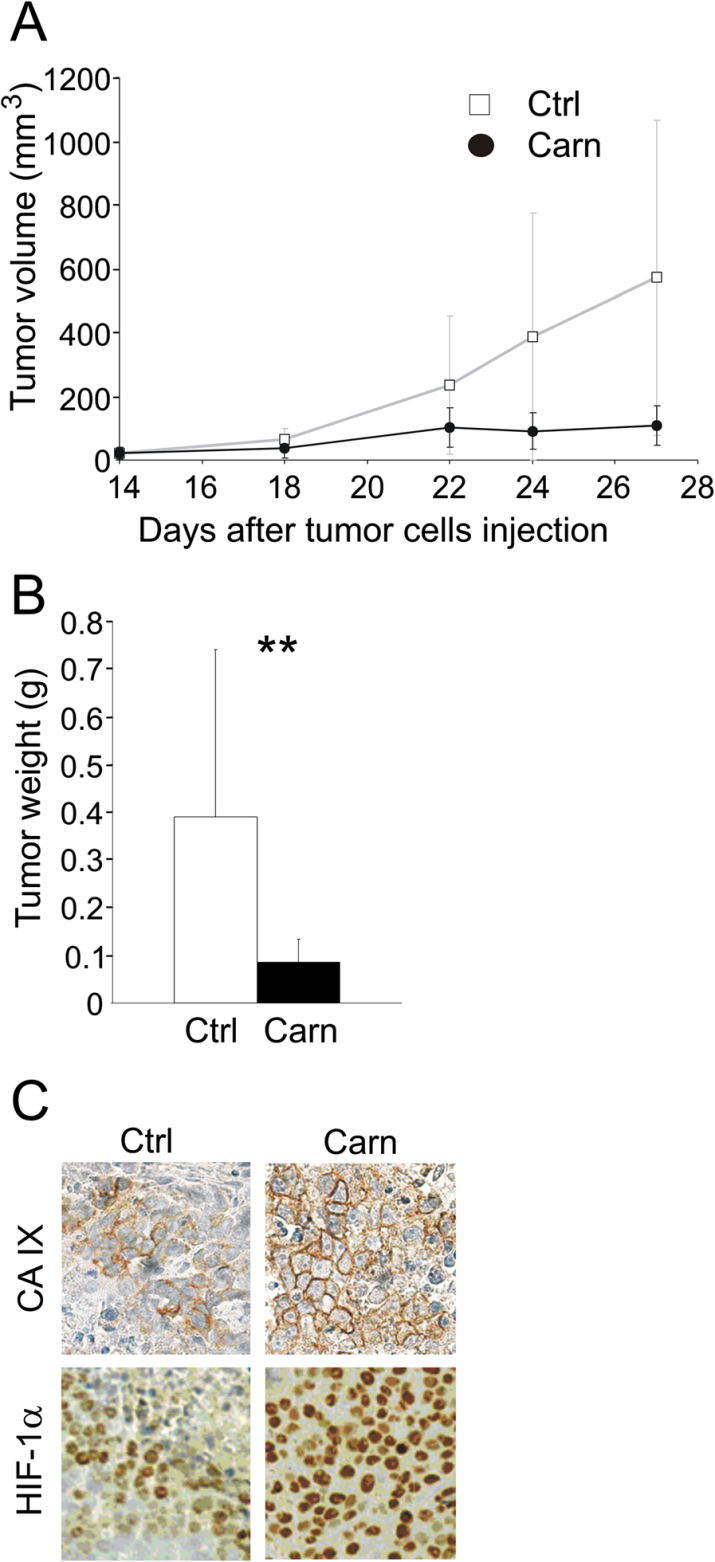
**Effect of carnosine in a mouse xenograft model. (A)** Graph showing slower growth of tumors in the group treated with carnosine. Carnosine treatment began on the 14^th^ day after subcutaneous implantation of HeLa cells, and tumor size was measured using a caliper during treatment. **(B)** There was a significant difference in the weight of tumors from treated and control groups (t-test, **p < 0.01). **(C)** Immunohistochemical staining of tumor tissue sections demonstrated enhanced expression of HIF-1α and CA IX proteins in the carnosine-treated group.

Tumor growth was visible 7 days after subcutaneous implantation of HeLa cells in all animals. On the 14^th^ day of the experiment, we separated the mice into two groups and started subcutaneous administration of carnosine solution to animals in the carnosine group. At the same time, we commenced caliper measurement of the tumors. All animals had comparable-sized tumors at the start of carnosine treatment. Between the 21^st^ and 28^th^ day of the experiment we noticed faster growth of tumors in the control group compared with the carnosine-treated group, in which the average tumor size remained relatively constant. Although several tumors continued to grow in the carnosine-treated group, the rate of tumor growth was very slow, whereas the growth of some tumors stopped and several tumors even became smaller (Figure 6A). These observations were confirmed after the final examination, when we found a significant difference in the weight of tumors between the control and carnosine-treated groups (Figure 6B). Immunohistochemical staining of formalin-fixed, paraffin-embedded tumor tissues demonstrated enhanced intensity of HIF1-α and CA IX staining in the carnosine-treated group (Figure 6C). These results correspond with the higher level of both proteins observed *in vitro*.

## Discussion

Identification of a potent anticancer drug that does not have adverse side effects remains a highly topical issue. The clinical use of carnosine in children with autism [27] and the use of a zinc complex of L-carnosine (Polaprezinc) as an anti-ulcer drug in Japan since 1994 have yielded encouraging results [28]. Application of this dipeptide in anti-cancer therapy is still an emerging field, but carnosine seems to be a promising candidate because of its anti-tumorigenic effects combined with its natural presence in the body and its beneficial influence on normal cells. These positive effects include protection of astroglial cells by NO trapping [29], protection against hypoxia-ischemia brain damage [30], reduction in the development of inflammation and tissue injury associated with spinal cord trauma [31], protection of lung tissue against bleomycin-induced injury [32], prevention of vascular damage in experimental diabetic retinopathy [33], and protection against ionizing radiation [34,35].

There is a lack of published data on carnosine administration in human participants in the setting of cancer treatment. Two recently published articles report no adverse effects for carnosine dosages of 4 g as a single dose [36] or 400 mg twice daily [27]. These data indicate the feasibility of carnosine as a therapeutic agent, although more clinical trials are clearly needed. In this article, we focused on the effect of carnosine on the hypoxic pathway, which is often up-regulated in tumors, and specifically on its effect on CA IX, expression of which is often associated with solid hypoxic tumors. The major function of CA IX in cancer is maintenance of pH homeostasis, which is related to the acidification of the tumor microenvironment that promotes cancer cell migration and invasiveness. Our results showed that carnosine causes a shift in extracellular pH (pHe) from acidic to alkaline for various cancer cell lines (HeLa, SiHa, HT-29) under hypoxic conditions. Although carnosine functions as a physiologic buffer, the observed change in pHe cannot be attributed to its buffering capacity alone. pH measurements of mock-transfected and CA IX-transfected MDCK cells demonstrated that carnosine treatment leads to a reduction of CA IX acidification activity, indicating a direct influence of carnosine on CA IX function.

Transcription of *CA9* is induced by the binding of HIF-1α to its core promoter [23]. Available information on the effect of carnosine on HIF-1α is very limited. Using a gene reporter assay, Oppermann *et al*. found enhanced activity of HIF-1α in the presence of carnosine and hypoxic conditions in the glioblastoma cell lines T98G, LN405, and 1321N1 and in one primary culture [37]. In contrast, Bharadwaj *et al.* showed that carnosine treatment decreased HIF-1α expression in H9c2 cardiomyoblasts, but not in human astrocytes; however, neither of these cell lines is cancerous [17]. In our study, carnosine treatment increased protein and mRNA levels of HIF-1α, and its effect on HIF-1α activity was demonstrated by up-regulation of the target genes *CA9*, *VEGF,* and *GLUT1* through the HRE element in their promoters. Degradation of HIF-1α protein is mediated by the VHL tumor-suppressor protein, which interacts with von Hippel-Lindau binding protein 1 (VBP1) and forms a complex that is transported to the nucleus or cytoplasm. Proteomic studies of glioblastoma cells treated with carnosine showed significantly reduced expression of VBP1 protein and mRNA [15]. This is consistent with our findings that the level of *VBP1* mRNA decreased in hypoxic HeLa cell monolayers after treatment with carnosine. Hence, the increase in HIF-1α protein level after carnosine treatment could be a consequence of its stabilization due to reduced VHL-mediated degradation.

Hypoxia plays an important role in cancer progression and metastasis [38,39] and there is growing evidence that altered tumor metabolism and HIF-1-regulated enzymes such as CA IX and CA XII may be vital in the process of primary tumor progression to metastasis. Tumor-specific expression of CA IX and its association with tumor invasiveness and poor treatment outcome has led to interest in targeting this enzyme for cancer therapy [22,40]. CA IX activity plays an important role in the survival of tumor cells in hypoxic regions of solid tumors through the neutralization of intracellular pH and consequential acidification of the extracellular environment [26]. Because increased expression of HIF-1α and CA IX in various tumors is believed to be associated with poor prognosis of cancer patients, an understanding of their involvement in the induction of tumor cell proliferation and consequent tumor growth has clinical relevance.

Carnosine inhibits the growth of tumors formed from different neoplastic cell lines [11-14]. Consistent with these findings, we observed an approximately 50% decrease in the size of carnosine-treated HeLa spheroids and reduced growth of tumors in carnosine-treated animals. Our data from immunoblotting, flow cytometry, and immunofluorescence analyses showed a slight increase in CA IX protein level after carnosine treatment in accordance with the increase in HIF-1α, suggesting that the anti-tumor effect of carnosine cannot be explained by modulation of the amount of CA IX. Flow cytometric analysis showed that carnosine markedly reduced the viability of cells in hypoxic monolayers as well as in a 3D cellular model in which hypoxia naturally develops. The impaired viability of carnosine-treated cells is at least partially attributable to the effect of carnosine on CA IX catalytic activity and its implications for extracellular pH.

In conditions of hypoxia, and the consequent acidosis linked to high production of lactic acid, accurate regulation of intracellular pH (pHi) could represent a key process that allows a cell to escape damage induced by these unfavorable conditions. Importantly, a change in pHi of approximately 0.1 units could induce specific effects in several processes crucial for cell homeostasis, such as ATP production, cell proliferation, and protein synthesis.

Adaptation of tumor cells to hypoxia is a complex process involving many metabolic and regulatory pathways. The different effect of carnosine on normal and tumor cells might be associated with metabolic differences between these cells. Normal cells derive the maximum possible energy from glucose by oxidizing it completely to CO_2_ (32 moles ATP) and if an adequate oxygen supply is not available they use anaerobic glycolysis to form lactate as the end product (2 moles ATP). In contrast, tumor cells preferentially use the anaerobic pathway. It is possible that carnosine inhibits glycolytic metabolism prior to the formation of triose phosphate by stimulating the activity of fructose 1,6-biphosphate, thus creating a fruitless ATP-consuming cycle [13,41]. In support of this, the amount of ATP in a HeLa cell monolayer was markedly reduced after carnosine treatment in hypoxia (data not shown), and a similar mode of action was observed in studies on different cancer cell lines [14,42]. This depletion of ATP could result in reduced cell proliferation.

The fact that carnosine treatment resulted in a decreased ability of CA IX-expressing cells to acidify their extracellular environment indicates that carnosine affects CA IX catalytic function. This is supported by reduced binding of the homosulfanilamide CA inhibitor (CAI) to carnosine-treated cells. Fluorescein-conjugated CAI could bind only to hypoxic cells that expressed CA IX, evoking the idea that hypoxia induces catalytic activity of CA IX by modulating CA IX folding in a manner that opens the active site and makes it accessible to the inhibitor [26]. Other studies showing that binding of the inhibitor was markedly reduced after reoxygenation of cells also indicate that sulfonamide-based inhibitors accumulate on CA IX-positive cells only under hypoxic conditions [43]. Our results further support an interaction between carnosine and CA IX protein. The specific antibodies MAb10 and MAb12 used in this study react with conformational epitopes in the catalytic domain [25]. The results of competitive ELISA indicated that direct binding of carnosine to CA IX influenced CA IX conformation and reduced binding of these specific antibodies. A change in CA IX conformation might also affect interactions of CA IX with its protein partners in a metabolon and thus modulate CA IX activity. Indeed, impaired formation of a bicarbonate transport metabolon was demonstrated by a reduced signal in the proximity ligation assay between CA IX and AE2.

Although the application of carnosine in clinical settings, especially as an anti-neoplastic therapeutic, has been discussed for several years, experimental-based explanations of its effects are still insufficient and no double-blind clinical trials have been performed [44]. Nagai and Suda first described the anti-neoplastic effects of carnosine on Sarcoma-180 cells implanted subcutaneously into ddY mice [45]. Renner *et al.* showed that carnosine delays aggressive tumor growth in nude mice after subcutaneous implantation of cells expressing human epidermal growth factor receptor 2 by affecting proliferation *in vivo *[13]. They also demonstrated that carnosine inhibits growth of cells from human malignant glioma and identified carnosine as an inhibitor of anaerobic glycolysis that is vital for the growth of gliomas [42]. A recent study revealed that carnosine inhibited tumor proliferation of human colon cancer cells transplanted into athymic nude mice, probably by elevating natural killer activity of splenic cells [46]. Carnosine was also shown to inhibit KRAS-mediated HCT116 proliferation [14], to inhibit metastasis of SK-Hep-1 invasive hepatocarcinoma cells by inhibiting expression and activity of matrix metalloproteinase 9 [47], and to eliminate tumor cells from a mixture of normal fibroblasts and HeLa cells [12].

As CA IX expression is predominantly associated with tumors and is often a marker of poor prognosis, the possible inhibition of its activity by carnosine deserves further investigation because it could lead to anticancer activity without blocking the expression of HIF-1α. The fact that the binding of monoclonal antibodies to CA IX is not inhibited at physiologic concentrations of carnosine can be perceived as an advantage as it enables the use of immunotherapy with parallel blocking of CA IX function by carnosine. The induction of CA IX protein expression by carnosine could also be a positive side effect, as it creates better conditions for immunotherapy through increasing the number of target molecules.

## Conclusions

CA IX is often expressed in solid tumors and is considered a marker of hypoxia and an indicator of poor prognosis. Carnosine reduces the extracellular acidosis linked to catalytic activity of CA IX in hypoxia and inhibits the growth of spheroids and tumor xenografts. Our results suggest that the interaction of carnosine with CA IX leads to conformational changes of the CA IX protein and impairs formation of its metabolon, which in turn influences its function. Thus, carnosine could be a promising anticancer drug through its ability to attenuate the activity of CA IX.

## Competing interests

The authors declare that this research was conducted in the absence of any commercial or financial relationship that could be construed as a potential conflict of interest.

## Authors’ contributions

ZD and JP conceived and designed the experiments; ZD, PD, ML, FI, VS, MZ, and LC performed the experimental work and participated in data analysis; ZD, LC, and VS drafted the manuscript; JP and SP critically revised the manuscript and made many conceptual suggestions. All authors read and approved the final manuscript.

## Pre-publication history

The pre-publication history for this paper can be accessed here:

http://www.biomedcentral.com/1471-2407/14/358/prepub

## Supplementary Material

Additional file 1 **SiHa, HeLa, HT-29, MDCK CA IX and MDCK mock cells were exposed to hypoxia for 48 hours in the presence of 20 mM carnosine.** The columns in the graph represent endpoint values of pH of carnosine treated cells and their respective untreated controls. Carnosine caused a considerable alkalization of growth media in all studied cell lines, except for MDCK mock cells where the effect of carnosine was noticeably lower. Differences between control and carnosine treated cells were evaluated by t-test (*p < 0.05, **p < 0.01). Click here for file

Additional file 2 **Western blot representing the expression level of total CA IX protein and its phosphorylated form at threonine 443 in the intracellular tail in HeLa cells cultured in hypoxia with and without 20 mM carnosine.** Numbers underneath the protein bands give the increase in the signal after carnosine treatment compared to untreated cells. Carnosine did not cause increased phosphorylation of T443 as the increase of levels of pT433 and total CA IX is comparable. Click here for file
