# Supplementary figures and images for: Carnosine inhibits carbonic anhydrase IX-mediated extracellular acidosis and suppresses growth of HeLa tumor xenografts
Source: BMC Cancer. 2014 May 22;14:358. doi: 10.1186/1471-2407-14-358 (PMC4061103; doi:10.1186/1471-2407-14-358)

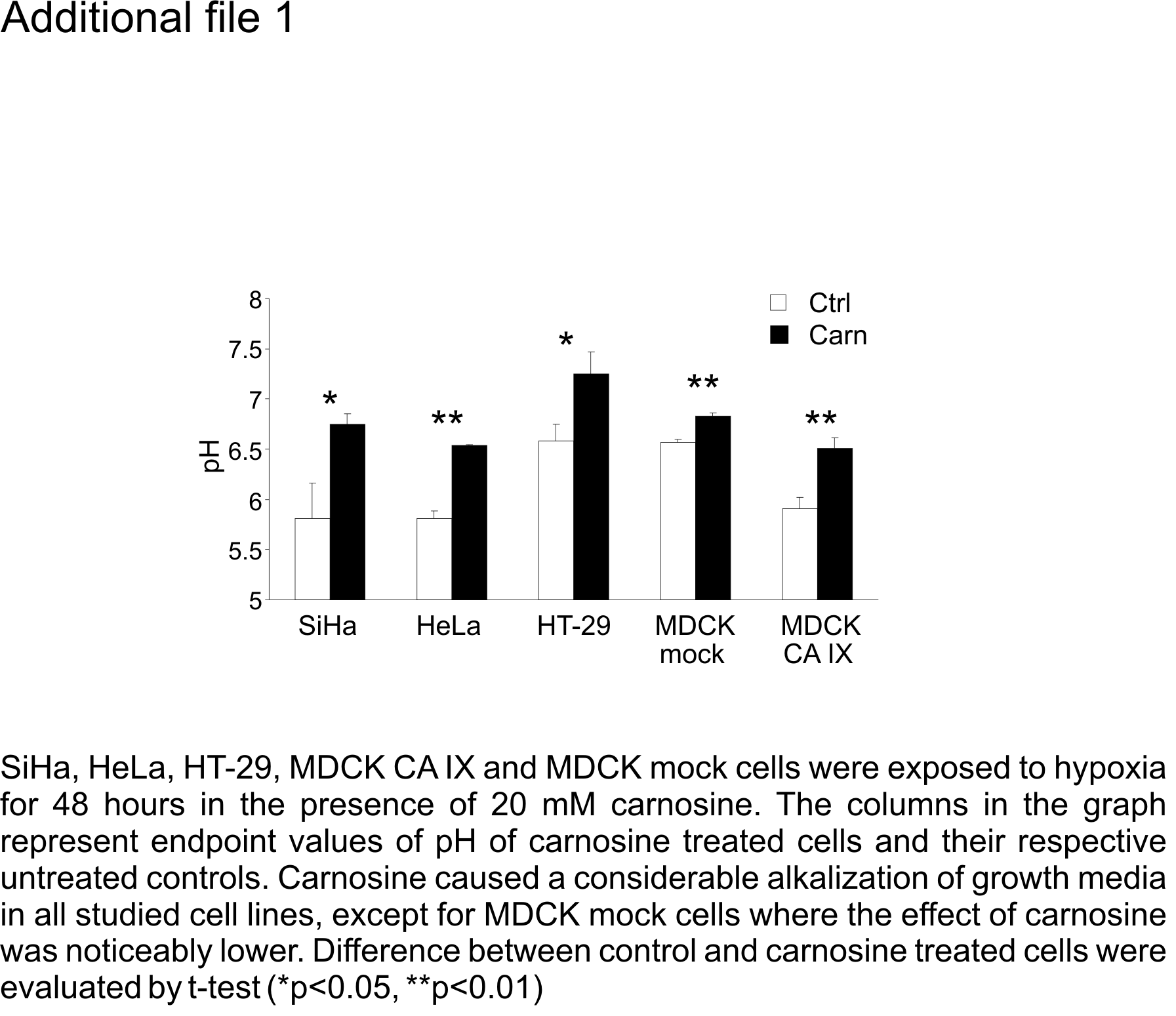

Supplement: Additional file 1 — SiHa, HeLa, HT-29, MDCK CA IX and MDCK mock cells were exposed to hypoxia for 48 hours in the presence of 20 mM carnosine. The columns in the graph represent endpoint values of pH of carnosine treated cells and their respective untreated controls. Carnosine caused a considerable alkalization of growth media in all studied cell lines, except for MDCK mock cells where the effect of carnosine was noticeably lower. Differences between control and carnosine treated cells were evaluated by t-test (*p < 0.05, **p < 0.01). [file 1471-2407-14-358-S1.tif]

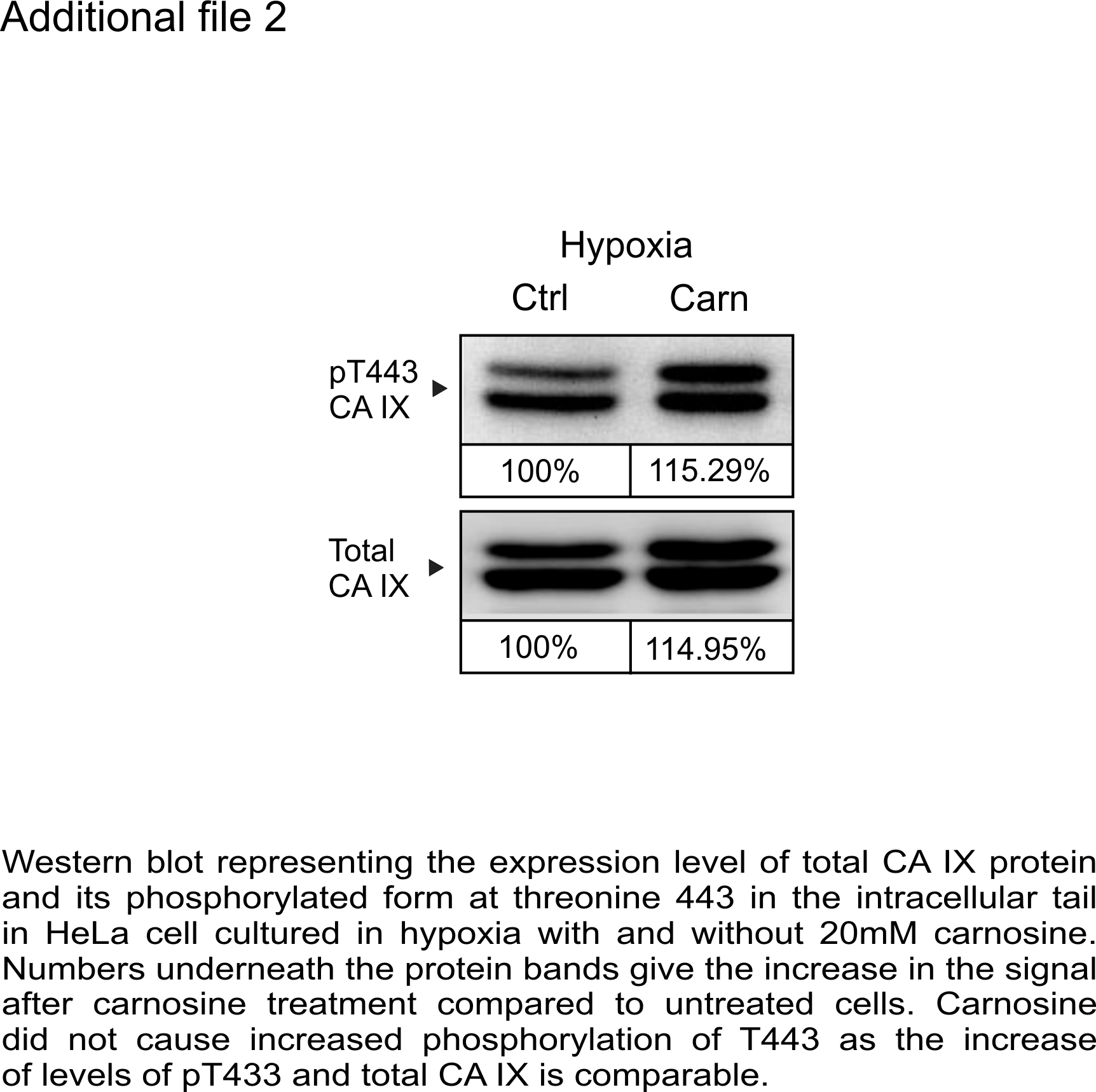

Supplement: Additional file 2 — Western blot representing the expression level of total CA IX protein and its phosphorylated form at threonine 443 in the intracellular tail in HeLa cells cultured in hypoxia with and without 20 mM carnosine. Numbers underneath the protein bands give the increase in the signal after carnosine treatment compared to untreated cells. Carnosine did not cause increased phosphorylation of T443 as the increase of levels of pT433 and total CA IX is comparable. [file 1471-2407-14-358-S2.tif]
